# Different types of mesh fixation for laparoscopic repair of inguinal hernia: A protocol for systematic review and network meta-analysis with randomized controlled trials

**DOI:** 10.1097/MD.0000000000010423

**Published:** 2018-04-20

**Authors:** Kongyuan Wei, Cuncun Lu, Long Ge, Bei Pan, Huan Yang, Jinhui Tian, Nong Cao

**Affiliations:** a Department of General Surgery, First Affiliated Hospital of Lanzhou University; b Evidence-based Medicine Center, School of Basic Medical Sciences; c Department of Social Medicine and Health Management, School of Public Health, Lanzhou University, Lanzhou, China.

**Keywords:** inguinal hernia, laparoscopic, mesh fixation, network meta-analysis

## Abstract

**Background:**

Laparoscopic inguinal hernia repair has become a valid option for repair of an inguinal hernia. Due to there are several types of mesh fixation for laparoscopic repair of inguinal hernia. The study aims to assess and compare the efficacy of different types of mesh fixation for laparoscopic repair of inguinal hernia using network meta-analysis.

**Methods:**

We will systematically search PubMed, EMBASE the Cochrane library, and Chinese Biomedical Literature Database from their inception to March 2018. Randomized controlled trials (RCTs) that compared the effect of different types of mesh fixation for laparoscopic inguinal hernia repair will be included. The primary outcomes are chronic groin pain, incidence risk of hernia recurrence, and complications. Risk of bias assessment of the included RCTs will be conducted using to Cochrane risk of bias tool. A network meta-analysis will be performed using WinBUGS 1.4.3 software and the result figures will be generated using R x64 3.1.2 software and STATA V.12.0 software. Grading of Recommendations Assessment, Development and Evaluation (GRADE) will be used to assess the quality of evidence.

**Results:**

The results of this study will be published in a peer-reviewed journal.

**Conclusion:**

Our study will generate evidence of laparoscopic repair of mesh fixation for adult patients with inguinal hernia and provide suggestions for clinical practice or guideline.

## 1 Introduction

The inguinal hernia is a protrusion of abdominal contents into the inguinal canal through an abdominal wall defect,^[1]^ and the risk of inguinal hernia increases with age, from 0.25% at 18 years of age to 4.2% at 75 to 80 years of age.^[2]^ In China, about 2,000,000 inguinal hernias are diagnosed each year.^[3]^ Inguinal hernia is one of the most commonly encountered conditions in surgical practice, and the surgical treatment of inguinal hernia can be achieved via an open or a minimally invasive laparoscopic approach.^[4]^ Moreover, approximately 800,000 are performed with surgery each year in the United States.^[5]^

Currently, inguinal hernia repair with a mesh is the mostly common method through surgical procedure. Among the surgical risk factors are the type of mesh and its fixation technique.^[6]^ The current type of mesh including different materials, and surgical options for mesh fixation include, but are not limited to, sutures, tacks or staples, self-fixing meshes and fibrin, or other glues.^[7]^ Chronic groin pain is the one of the main problems after surgery; however, laparoscopic techniques have had better results in chronic groin pain.^[8]^ Besides, there is a continuing increase in the number of laparoscopic procedures performed since their introduction using mesh in the late 1991.^[9]^

Now, laparoscopic inguinal hernia repair has become a valid option for repair of an inguinal hernia. To our best of knowledge, though previous meta-analysis^[10]^ compared efficacy of different types of mesh fixation methods for ventral hernia during laparoscopic repair, there is no network meta-analysis for comprehensive comparison to assess different types of mesh fixation methods in laparoscopic repair for inguinal hernia and this will be the first network meta-analysis to compare all of mesh fixations for laparoscopic repair of inguinal hernia with RCTs. Therefore, we will conduct a systematic review and network meta-analysis to explore which mesh fixation for laparoscopic repair of inguinal hernia is the potentially optimal method and provide recommendations for surgeon.

Network meta-analysis has been considered to extend conventional meta-analyses on multiple treatments (i.e., 3 or more) for a given condition.^[11]^ Hence, it becomes increasingly popular to evaluate healthcare interventions, since it allows for estimation of the relative effectiveness among all interventions and rank ordering of the interventions even if head-to-head comparisons are lacking.^[12]^ Trial sequential analysis (TSA) is a tool for quantifying the statistical reliability of the data in a cumulative meta-analysis^[13,14]^ and we will perform TSA to explore pooled data.

## 2 Methods

### 2.1 Eligibility criteria

#### 2.1.1 Type of study

Randomized controlled trials (RCTs) that compared the effect of different types of mesh fixation for laparoscopic inguinal hernia repair will be included. We will include RCTs reported in any language.

#### 2.1.2 Type of patients

We will include adults (aged 18 years or older) with inguinal hernia, who scheduled for laparoscopic inguinal hernia repair.

#### 2.1.3 Type of interventions

We will include studies that reported different mesh fixation methods (or fixation vs no fixation) in laparoscopic inguinal hernia repair, including, but not limited to, sutures, tacks or staples, self-fixing meshes and fibrin, or other glues.

#### 2.1.4 Type of outcomes

The primary outcomes are chronic groin pain, incidence risk of hernia recurrence and complications. The secondary outcomes include operative time, length of hospital stay, and postoperative pain. Chronic groin pain is groin pain persisting at least 3 months after the index operation, recurrence was defined as clinical or radiologic recurrence of inguinal hernia, complications was defined as any complications requiring further procedures in the theatre during the same surgical admission, operative time was defined as time from skin incision to skin closure, length of hospital stay was defined as time from the index operation to discharge and postoperative pain was defined as VAS (visual analog scale) immediately after and during 1 week of the operation. RCTs reporting on at least one related outcome will be included.

### 2.2 Data source

We will systematically search PubMed, EMBASE the Cochrane library, and Chinese Biomedical Literature Database from their inception to March 2018. Search strategy of PubMed was as follows:

#1 “Hernia, Inguinal”[Mesh]

#2 “Surgical Mesh”[Mesh]

#3 groin hernia[Title/Abstract]

#4 inguinal hernioplasty[Title/Abstract]

#5 mesh[Title/Abstract]

#6 inguina∗ AND hernia[Title/Abstract]

#7 or/1–6

#8 “Randomized Controlled Trial” [Publication Type] OR “Randomized Controlled Trials as Topic”[Mesh] OR “Controlled Clinical Trial” [Publication Type]

#9 random∗[Title/Abstract] OR “clinical trial∗”[Title/Abstract]

#10 or/8–9

#11 #7 and #10

### 2.3 Study selection

All authors involved in this study had previous experience of completing systematic reviews. We will use EndNote X7 to manage citations from databases. The title and abstract of each citation retrieved will be checked by 2 independent reviewers (KW and CL) according to eligibility criteria. The full texts of potentially relevant studies will be retrieved for further assessment. Disagreements will be resolved by discussion or consultation of a 3rd author (LG). We will use predefined extraction forms with detailed written instructions which will be will be created using Microsoft Excel 2013 to collect relevant information and data. The information will include first author, year of publication, sample size, interventions, and outcomes, and the third reviewer to check information and data. Study selection and information extraction will conduct formal calibration exercises with relevant reviewers before the research start. when some studies report median rather than mean, and range or interquartile range rather than SD (standard deviation), in which case the mean and SD will be estimated.^[15]^

### 2.4 Risk of bias of individual studies

Two of reviewers independently used the Cochrane Handbook V.5.1.0 for systematic reviews of intervention to assess the quality of included RCTs,^[16]^ which was composed of 6 domains: random sequence generation, allocation concealment, blinding of all participants, including patients, personnel and outcome assessors, incomplete outcome data, selective reporting, and other source of bias. We will evaluate methodological quality as low, high or unclear risk of bias.

### 2.5 Dealing with missing data

We will not contact authors to obtain missing information of primary studies. If binary outcomes are missing, we will perform an available-case analysis, but we will assess the impact of “best-best,” “best-worst,” “worst-best,” and “worst-worst” scenario analyses.^[17]^

### 2.6 Statistical analysis

#### 2.6.1 Trial sequential analysis

We will carry out TSA to reduce random errors.^[14]^ TSA will be performed for dichotomous outcomes as well as for continuous outcomes to control the risks of random errors due to sparse data and multiplicity.^[18]^

#### 2.6.2 Pairwise meta-analyses

The pairwise meta-analyses will be performed using random-effects model by R x64 3.1.2 software. The odds radio (OR) with 95% confidence interval (95% CI) will be used to measure dichotomous outcomes (including hernia recurrence, complications) and the mean difference (MD) with 95%CI will be presented for continuous data (operative time, length of hospital stay, and postoperative pain). Assuming that treatment effect would vary across studies due to both sampling variability and other factors such as differences in surgical skill and the numbers of procedures carried out by a surgeon, random effects model was used to pool effect estimates. The potential heterogeneity across the included studies was tested using *I*^2^. If the *P* value ≥.1 and *I*^2^ is ≤50%, it suggests that there is no statistical heterogeneity, and the Mantel–Haenszel fixed effects model will be used for meta-analysis. If the *P* value < .1 and *I*^2^ is >50%, we will explore sources of heterogeneity by subgroup analysis and meta-regression. Publication bias will be examined using Begg's and Egger's funnel plot method through STATA V.12.0 software (Stata Corporation, CollegeStation, Texas) when at least included 10 studies for one related outcome.^[19]^

#### 2.6.3 Network meta-analyses

A Bayesian NMA will be performed by WinBUGS 1.4.3 software (MRC Biostatistics Unit, Cambridge, UK).We will use node splitting method to examine the inconsistency between direct and indirect comparisons if a loop connecting 3 or more arms exist.^[20]^ Surface under the cumulative ranking area (SUCRA) will be used to rank the different types of mesh fixation, a larger surface under the cumulative ranking means a more effective intervention.^[21]^ Comparison-adjusted funnel plots will be conducted to assess the effects of the sample size on the results. A network plot will be drawn to describe and present the geometry of the treatment network of comparisons across trials to ensure if a network meta-analysis is feasible. Trials will be excluded if the trials are not connected by treatments. Network geometry will use nodes to represent different interventions and edges to represent the head-to-head comparisons between interventions. The size of nodes and thickness of edges are associated with sample sizes of intervention and numbers of included trials, respectively. All the result figures will be generated using R × 64 3.1.2 software.

### 2.7 Quality of evidence

The quality of evidence for the primary outcomes will be assessed using the Grading of Recommendations Assessment, Development and Evaluation (GRADE),^[22]^ according to the comprehensive result of factors (risk of bias, inaccuracy, inconsistency, indirectness, publication bias) that influenced evidence quality which grades 4 levels: High level, moderate level, low level, and very low level.

## 3 Discussion

To the best of our knowledge, this is the first network meta-analysis protocol comparing different types of mesh fixation methods to repair laparoscopic inguinal hernias with RCTs. The study will provide a ranking of mesh fixation for laparoscopic inguinal hernias and we hope the result will provide recommendations for repairing of laparoscopic inguinal hernias This protocol is designed in adherence to guideline for network meta-analysis protocols and will be conducted and reported strictly according to the PRISMA extension statement for network meta-analysis.^[23]^ Recent international guidelines for hernia management^[24,25]^ provide mounts of suggestions for clinical practice and we will focus on the controversial problems to explore the favor options.

## Author contributions

WKY, LCC, and GL plan and design the research; WKY, TJH and YH tested the feasibility of the study; WKY, LCC and CN provided methodological advice, polished and revised the manuscript; WKY and PB wrote the manuscript; all authors approved the final version of the manuscript.

**Data curation:** Bei Pan, Huan Yang.

**Formal analysis:** Jinhui Tian.

**Investigation:** Huan Yang.

**Methodology:** Cuncun Lu, Long Ge.

**Software:** Long Ge, Jinhui Tian.

**Writing – original draft:** Kongyuan Wei, Nong Cao.

**Writing – review & editing:** Kongyuan Wei, Nong Cao.
